# ContextD: an algorithm to identify contextual properties of medical terms in a Dutch clinical corpus

**DOI:** 10.1186/s12859-014-0373-3

**Published:** 2014-11-29

**Authors:** Zubair Afzal, Ewoud Pons, Ning Kang, Miriam CJM Sturkenboom, Martijn J Schuemie, Jan A Kors

**Affiliations:** Department of Medical Informatics, Erasmus Medical Center, P.O. Box 2040, Rotterdam, CA 3000 Netherlands; Janssen Research and Development LLC, Titusville, NJ USA

**Keywords:** Contextual features, Negation detection, Dutch electronic medical records

## Abstract

**Background:**

In order to extract meaningful information from electronic medical records, such as signs and symptoms, diagnoses, and treatments, it is important to take into account the contextual properties of the identified information: negation, temporality, and experiencer. Most work on automatic identification of these contextual properties has been done on English clinical text. This study presents ContextD, an adaptation of the English ConText algorithm to the Dutch language, and a Dutch clinical corpus.

We created a Dutch clinical corpus containing four types of anonymized clinical documents: entries from general practitioners, specialists’ letters, radiology reports, and discharge letters. Using a Dutch list of medical terms extracted from the Unified Medical Language System, we identified medical terms in the corpus with exact matching. The identified terms were annotated for negation, temporality, and experiencer properties. To adapt the ConText algorithm, we translated English trigger terms to Dutch and added several general and document specific enhancements, such as negation rules for general practitioners’ entries and a regular expression based temporality module.

**Results:**

The ContextD algorithm utilized 41 unique triggers to identify the contextual properties in the clinical corpus. For the negation property, the algorithm obtained an F-score from 87% to 93% for the different document types. For the experiencer property, the F-score was 99% to 100%. For the historical and hypothetical values of the temporality property, F-scores ranged from 26% to 54% and from 13% to 44%, respectively.

**Conclusions:**

The ContextD showed good performance in identifying negation and experiencer property values across all Dutch clinical document types. Accurate identification of the temporality property proved to be difficult and requires further work. The anonymized and annotated Dutch clinical corpus can serve as a useful resource for further algorithm development.

**Electronic supplementary material:**

The online version of this article (doi:10.1186/s12859-014-0373-3) contains supplementary material, which is available to authorized users.

## Background

Recent years have seen an increase in the use of electronic medical records (EMRs) by healthcare providers [1]. These records contain patient-related information such as signs, (patient-reported) symptoms, diagnoses, treatments, and tests. The primary use of EMRs is to support the care process, but the secondary use of EMRs for clinical research is increasing. In most EMRs, the majority of information is unstructured free text, making information retrieval challenging, although several automatic systems have been developed that can index, extract, and encode clinical information from the EMRs [2-8]. One particular challenge in analyzing free-text EMRs is to distinguish positive diagnoses by the physician from things that have been excluded or ruled out. Similarly, information about the past medical problems and also a family history is often found in the EMRs and should ideally be identified as such. In order to extract meaningful information such as medical problems or clinical conditions, it is important that automatic systems do not only identify them but also take into account the context of the identified information.

Previous approaches on identifying contextual properties of clinical concepts can be classified into rule or regular-expression based techniques, machine-learning techniques, or a combination of both. Chapman et al. [9] developed a rule-based system called NegEx that determines whether a specific medical condition is present or absent within a narrative. The system uses two sets of trigger phrases: one to identify true negations and a second to identify pseudo-negations, i.e., phrases that seem to indicate negation but instead denote double negations such as *not ruled out*. The system was evaluated on discharge summaries where it achieved a precision of 84.5% and a recall of 77.8%. Another system, called NegFinder [10], used grammatical parsing and regular expressions to identify negated patterns occurring in medical narratives, achieving a specificity of 97.7% and a sensitivity (or recall) of 95.3% on discharge summaries and surgical notes. Elkin et al. [11] assigned a level of certainty to identified concepts in EMRs based on a rule-based system to decide whether a concept has been positively, negatively, or uncertainly asserted. The system achieved 97.2% sensitivity, 98.8% specificity, and 91.2% precision on medical evaluation notes. Huang et al. [12] used regular expressions with grammatical parsing to identify negated phrases. On radiology reports, the system achieved a sensitivity of 92.6%, a specificity of 99.8%, and a precision of 98.6%. The ConText algorithm [13] is based on the NegEx algorithm and apart from identifying negations it also identifies whether a clinical condition is present, historical, or hypothetical, and whether the clinical condition is experienced by the patient or someone else, e.g., a family member. The system achieved an average precision of 94% and an average recall of 92% when evaluated on six different types of medical reports. Kilicoglu and Bergler [14] showed that speculative language can be recognized successfully using linguistically oriented approaches. They extended lexical resources with syntactic patterns and introduced a simple weighting scheme to estimate the speculation level of the sentences. The system achieved a precision of 85% and a recall of 86%. Recently, Reeves et al. [15] created a system, Med-TTK, to identify and classify temporal expressions in medical narratives. The system achieved a precision of 85% and a recall of 86% on clinical notes.

In machine-learning approaches, Goldin and Chapman [16] experimented with Naïve Bayes and decision trees to determine whether a concept is negated by the word *not* in hospital progress notes and emergency room notes. Agarwal and Yu [17] used conditional random fields (CRF) to detect negation cues and their scopes. The best CRF model achieved a precision of 99% and a recall of 96% on detecting negation cues, and a precision and recall of 95% on detecting their scopes in clinical notes. Morante and Daelemans [18] first used a classifier to identify negation signals and then used four classifiers to find the full scope of the negation signals. Three of the classifiers predicted whether a token was the first, the last, or neither in the scope sequence. The fourth classifier was a meta-learner that used the prediction of first three classifiers to determine the final scope. On BioScope clinical documents [19], the system achieved a precision of 86% and a recall of 82%, and 71% of negation scopes were correctly identified. Cruz Díaz et al. [20] improved on Morante and Daelemans [18] by using different classifiers. The system achieved a precision of 92%, a recall of 90%, and 88% of the negation scopes were correctly identified. To detect speculation, the system achieved a precision of 85%, a recall of 63%, and 63% of speculation scopes were correctly identified. Light et al. [21] estimated that 11% of the sentences in MEDLINE abstracts contain speculative fragments. They used a substring matching method and Support Vector Machines (SVM) to determine whether concepts in the text are described as facts or as speculation. For the matching method, they identified 14 strings that suggest speculation and marked a sentence as speculative if their system found any of these strings in the sentence (possibly as a substring of a term). The SVM classifier achieved a precision of 84% and a recall of 37% whereas substring matching achieved a precision of 55% and a recall of 79%. Velldal [22] used a disambiguation approach and SVM-based classifiers to label sentences as certain or uncertain. Their best system achieved a precision of 89% and a recall of 85%. Goryachev et al. [23] compared two adaptations of regular-expression based algorithms, NegEx and NegExpander, with two classification methods, Naïve Bayes and SVM, trained on discharge reports. It was observed that regular-expression based methods show better accuracy than the classification methods. Uzuner et al. [24] developed a statistical assertion classifier, StAC, by using lexical and syntactic context in conjunction with SVM to classify medical problems in EMRs into four categories: positive, negative, uncertain, and alter-association. StAC was compared to an extended version of the NegEx algorithm and showed better performance. The 2012 i2b2 NLP Shared Task [25] focused on finding the temporal relations in clinical narratives. While machine-learning and rule-based systems showed good performance, the systems using combination approaches produced the best results.

The type of clinical documents has a noticeable impact on the performance of systems that identify contextual properties of clinical concepts. Clinical documents differ in many ways, such as structure, grammaticality, and use of standard and non-standard abbreviations. Overall there does not seem to be a clear winner between machine-learning and rule-based systems. The rule-based and hybrid systems appear to perform slightly better than machine-learning approaches. In theory, rule-based systems can be adapted rather easily for different clinical text than for which they were developed. One of the limiting factors of a rule-based approach is the use of a fixed scope which may lead to misclassification. The machine-learning based approaches may not perform as well if they are tested on a different clinical text than they were originally trained on [23]. Adapting such approaches for new clinical text will therefore require a new training set.

Most work on identifying contextual properties of the clinical condition has been done on the English language. Recently, the NegEx algorithm was adapted to detect negations in Swedish [26] and French [27] clinical text. To our knowledge, no method is yet available or adapted for Dutch clinical text.

This study has two objectives: to adapt the well-known ConText [13] algorithm (to detect contextual properties of medical terms) to the Dutch language and to create a Dutch clinical corpus which is annotated for negation, temporality, and experiencer. ConText, along with its predecessor NegEx, is one of the most widely used algorithms in the field. It was chosen for its simplicity, ease of adaptability, and proven good performance on various types of English clinical text. The adapted ConText algorithm, dubbed ContextD, and the anonymized Dutch clinical corpus described here will be made publicly available for research purposes [28].

## Methods

This section provides details of the Erasmus Medical Center (EMC) Dutch clinical corpus annotated for the three contextual properties negation, temporality, and experiencer. We also describe the original ConText algorithm and its adaptation to the Dutch language.

### EMC Dutch clinical corpus

The anonymized corpus includes four types of clinical documents to capture different language use in the Dutch clinical setting.

- **General Practitioner entries [GP]**
  This set consists of entries from the IPCI database [29], a longitudinal collection of EMRs from Dutch general practitioners (GP) covering more than 1.5 million patients throughout the Netherlands. Each entry in the IPCI database pertains to a patient visit to the GP. These entries are not always grammatically well-formed text, and often follow the well-known SOAP structure (Subjective, Objective, Assessment, and Plan) [30]. The resulting database contains a broad range of information, including indications and following prescriptions for therapy, referrals, hospitalization and laboratory results. The structured information, such as diagnosis codes, is stored in a tabular format and the unstructured information is stored as free-text. Only the unstructured free-text was included in the corpus.
- **Specialist letters [SP]**
  These are letters written by a medical specialist – for example a cardiologist – and they are also procured from the IPCI database. The purpose of these letters is to report back to the GPs after referral and consult in the hospital, updating them in relation to diagnostic deliberations and therapeutic strategies. These letters are in the form of scanned copies or summaries entered by the GP. These letters are also not always grammatically well-formed.
- **Radiology reports [RD]**
  This set consists of the reports taken from the radiology department of the Erasmus Medical Center, The Netherlands. These reports contain descriptions and conclusions derived from diagnostic imaging as requested by medical specialists (doctors) or general practitioners. These reports are intended for communication between doctors and radiologists. The text is mostly generated by using an automatic speech recognizer (ASR) and therefore usually has proper grammar and structure by prevailing conventions of the Radiology department. The radiologists have the option to manually update the text generated by the ASR which increases the probability of typos.
- **Discharge letters [DL]**
  This set consists of patient discharge letters taken from the Erasmus Medical Center. They serve a purpose comparable to the specialist letters in updating the GPs on everything that has occurred during the admission period including all outcomes and remaining problems. These letters are well-formed because of their intended external use (by and beyond GPs) and continuity of care.

To select text from the above mentioned sets, we first created a list of Dutch medical terms taken from the Unified Medical Language System (UMLS) [31]. The UMLS contains medical terms in 21 different languages, including Dutch. However, UMLS has limited coverage of terms in the non-English languages. From over 150 source vocabularies in the UMLS, only four contain Dutch language terms. Only UMLS terms belonging to one of 35 UMLS semantic types, mainly representing diseases, symptoms, and drugs, were included in the list. A list of semantic types is presented in Additional file 1. The final term list contains 153,573 Dutch terms, including synonyms and lexical variants that were present in the UMLS. For each of the four sets, documents containing at least one UMLS term were randomly selected to be included in the corpus. We used case-insensitive exact string matching to find the UMLS terms in the documents. Table 1 summarizes the characteristics of the four document types.

**Table 1:** **Statistics of the four document types in the EMC clinical corpus**

**Type**	**No. of documents**	**No. of recognized UMLS terms**	No. of words per document*	
GP entries	2000	3626	23	(14–38)
Specialist letters	2000	2748	39	(16–113)
Radiology reports	1500	3684	66	(46–94)
Discharge letters	2000	2830	163	(95–201)

*Median (interquartile range).

Each of the recognized terms in the corpus was annotated for the three contextual properties: negation, temporality, and experiencer. The definitions of the properties are adopted from the ConText algorithm [13].

- Negation
  This property has two values, ‘Negated’ or ‘Not negated’. A clinical condition or term is labeled as ‘Negated’ if there is evidence in the text suggesting that the condition does not occur or exist, e.g., ‘There was no sign of sinus infection’, otherwise it is ‘Not negated’.
- Temporality
  The temporality property places a condition along a time line. There are three possible values for this property: ‘Recent’, ‘Historical’, and ‘Hypothetical’. A condition is considered ‘Recent’ if it is maximally 2 weeks old. Conditions that developed more than 2 weeks ago are labeled as ‘Historical’. A condition is labeled as ‘Hypothetical’ if it is not ‘Recent’ or ‘Historical’, e.g., ‘patient should return if she develops fever’ [13].
- Experiencer
  Clinical text may refer to subjects other than the actual patient. The experiencer property describes whether the patient experienced the condition or someone else. For simplicity, we have defined only two possible values for this property: ‘Patient’ or ‘Other’, where ‘Other’ refers to anyone but the actual patient, e.g., ‘Mother is recently diagnosed with cancer’.

The corpus was annotated by two independent annotators. They were provided with a guideline explaining the process and each of the contextual properties in detail, with examples. An expert who was familiar with all four types of clinical text resolved the differences between the annotators. The annotations were limited to the conditions previously identified using our custom Dutch UMLS terms. In The Netherlands, retrospective research with anonymized patient data does not fall under the scope of the WMO (Wet medisch-wetenschappelijk onderzoek met mensen (“Medical research involving human subjects act”)), and does not have to be approved by a medical ethics committee For the IPCI data, the access was approved by the IPCI governance board (Raad van Toezicht).

We split each of the four document sets in our corpus into a development set and an evaluation set (50% each). The development set was used to tune the algorithm and the trigger lists. To account for possible overfitting of the algorithm on the development set, the performance of the algorithm was assessed on the evaluation set, which was used only for the final testing.

#### The ConText algorithm

The ConText algorithm [13], an extension of NegEx [9], is based on regular expressions and lists of trigger terms to determine the values of three contextual properties of a clinical condition: negation, temporality, and experiencer. The algorithm searches a sentence for triggers before or after the pre-indexed clinical condition. The default value of a property (‘Not-negated’ for negation, ‘Recent’ for temporality, ‘Patient’ for experiencer) is changed if the condition falls within the scope of the trigger term. The default scope of a pre-trigger is from the right of trigger term to the end of the sentence, whereas the default scope of a post-trigger begins leftwards from the trigger term to the beginning of the sentence. The default scopes are overruled if a termination trigger is found before the end of the scope. For each property value (other than the default), the ConText algorithm maintains four lists of triggers: pre-triggers, post-triggers, termination triggers, and pseudo-triggers. Pre-triggers precede the location of a clinical condition in the text, e.g., *no signs of viral infection*. In this example, *viral infection* is the clinical condition and *no signs of* is the pre-trigger. Post-triggers follow a clinical condition, e.g., *viral infection is ruled out*. In this example, *ruled out* is a post-trigger. In both of these examples, the condition *viral infection* will be negated because it falls within the scope of the pre- and post-triggers. Termination triggers limit the scope of a pre- or post-trigger. Finally, there are phrases that look like triggers but do not act as such, e.g., *no change*. These are added to a pseudo-trigger list. The input to the algorithm is a sentence with marked clinical conditions. First, default values are assigned to the contextual properties of each clinical condition. The default values are then updated using the following algorithm:

- Find all trigger terms (pre, post, pseudo, termination) in the sentence
- For each of the trigger terms found (from left to right)
  - ○ If the term is a pseudo term, skip to the next term
  - ○ Otherwise:
  - ▪ Find the scope of the trigger term
  - ▪ Assign appropriate contextual property values to all marked clinical conditions within the scope.

Several implementations of the ConText algorithm are available online [32].

#### ContextD: ConText for Dutch

The ConText algorithm uses pre-defined English trigger terms to determine the value of the contextual properties. We first attempted a fully automated translation of these triggers into Dutch using Google Translate [33], but the results appeared not to be comprehensive enough. A native Dutch speaker, who was also familiar with clinical texts, then checked all automatically translated terms, and added all possible variations of a trigger term.

The ContextD algorithm expects a sentence with marked conditions as its input. We used the Dutch sentence splitter in the Apache OpenNLP library [34] to split the text into sentences. Using our custom UMLS Dutch term list and case-insensitive exact string matching, we marked all the UMLS terms in the sentences.

ContextD works like the original ConText algorithm in using the trigger lists to find the values of contextual properties. The Java implementation of ConText [32] with the translated triggers was used as a starting point. Using the development set, we iteratively refined the Dutch trigger lists and made a number of other modifications as described below:

#### GP specific rules

The general practitioners often negate the existence of a clinical condition by putting a minus sign after the term, e.g., *fever-.* We added a couple of rules to catch such occurrences (and their variations) of negation in the GP text.

#### Combined triggers

The value of a contextual property sometimes cannot be identified by a pre-trigger or a post-trigger alone, such as *nooit (never)* and *is weg (is gone)*. A similar weakness is also reported by Chapman et al. [9] for triggers *not* and *no*. For example, in the sentence ‘*Hij heeft verder nooit medicijnen gebruikt die de tinnitus beinvloeden (he has also never used medications that affect tinnitus)’,* the trigger *nooit* is negating the use of medication but not the condition *tinnitus.* Some of the triggers translated from the English cannot be directly applied to the Dutch text because of the different word ordering in both languages. Such triggers have to be split before they can be applied. There are situations where a combination of two triggers is essential. Since there is no notion of dependency or connection between different trigger types in the original ConText algorithm, we introduced a few rules that look for a combination of triggers to be present in order to identify the correct value of a contextual property. For example, in the sentence ‘*Nooit urineweginfecties doorgemaakt’,* the triggers *nooit (pre-trigger)* and *doorgemaakt (post-trigger)* combined suggest a negation for the term *urineweginfecties*. The pre-trigger *nooit* alone did not increase performance and hence was removed from the trigger list during the development.

#### Scope of trigger terms

ContextD uses different scopes depending on the trigger term. The default right-scope starts from the right of the trigger term and ends at the end of the sentence. The default left-scope starts leftwards from the trigger term and ends at the beginning of the sentence. We experimented with different scopes for different types of clinical text, which resulted in modifying the default scope for GP entries to 6 words and for specialist letters to 10 words. The default scope is overridden if a termination trigger appears before the end of the scope. For GP entries, which are mostly grammatically unstructured, some punctuation, such as comma and semicolon, were added as termination triggers to limit the scope of triggers. For specialist letters, only colon and semicolon were added to the termination triggers.

#### Temporality module

The original ConText algorithm has very few triggers to identify whether a clinical condition is historical. We added a temporality module that implements several regular expressions to look for evidence for historical events on both sides of the clinical term. An adjusted left and right scope was also implemented in the module to avoid getting false positives. The regular expressions used in the temporality module along with a brief description of the rules are presented in Additional file 1.

### Evaluation

We computed precision (true positives/[true positives + false positives]), recall (true positives/[true positives + false negatives]), and F-measure (the harmonic mean of precision and recall: 2 * precision * recall/[precision + recall]) for each of the three contextual properties.

For the negation property, terms that were assigned the value ‘Negated’ were taken as the positive class and terms that were marked ‘Not Negated’ as the negative class. Similarly, for the experiencer property, terms marked as ‘Patient’ were taken as positives, ‘Others’ as negatives. For the temporality property, which has three values, each value was considered as the positive class against the other two combined (e.g. ‘Recent’ vs. ‘Historical’ and ‘Hypothetical’). A true positive was defined as a term that was correctly assigned to the positive class, a false positive as a term that was incorrectly assigned to the positive class, and a false negative as a term that was incorrectly assigned to the negative class.

We used Cohen’s kappa [35] to calculate the agreement between both annotators for each of the three contextual properties. Because the UMLS terms were already marked in the sentences, the inter-annotator agreement was calculated for the labels only.

## Results

This section provides the annotation results of the EMC Dutch clinical corpus and the performance of the ContextD algorithm.

Table 2 shows the inter-annotator agreement for each report type in the corpus. According to the Altman classification [36], kappa is very good for ‘Negated’ and ‘Recent’ values (with the exception of ‘Recent’ on Radiology reports, which is good), moderate-to-good for ‘Historical’ values, and moderate for *hypothetical* values. The kappa for the *experiencer* property is very good except for in the radiology reports where a moderate agreement is observed. The lowest kappa score (moderate) of 0.46 is observed for the specialist letters for the value *hypothetical*. Because there was no hypothetical term in the discharge letters, no kappa was calculated for this property value.

**Table 2:** **Inter-annotator agreement on contextual properties in the EMC clinical corpus**

**Document type**	**Negated**	**Recent**	**Historical**	**Hypothetical**	**Patient**
GP entries	0.90	0.86	0.57	0.48	0.92
Specialist letters	0.90	0.93	0.62	0.46	0.98
Radiology reports	0.93	0.61	0.63	0.57	0.53
Discharge letters	0.94	0.95	0.56	n/a	0.98

Table 3 shows, for each report type, the distribution of the values of the three contextual properties. The distribution of the negated terms does not vary much between different report types. Historical conditions occur more frequently in specialists’ letters and discharge reports, 8% and 6% respectively, in comparison to GP entries (2%) and radiology reports (3%). This can be explained by the fact that specialist and discharge letters often include descriptions of the patients’ past medical history. Hypothetical terms are absent in discharge letters and infrequent (1% to 2%) in the other report types. The value *other* for the experiencer property is also found infrequently (from 0.1% to 2%) in all report types.

**Table 3:** **Distribution of the contextual property values in different types of clinical documents**

**Document type**	**Total**	**Negation**		**Temporality**			**Experiencer**	
		**Negated**	**Not-negated**	**Recent**	**Historical**	**Hypothetical**	**Patient**	**Other**
GP entries	3626	12%	88%	97%	2%	1%	98%	2%
Specialist letters	2748	15%	85%	90%	8%	2%	99%	1%
Radiology reports	3684	16%	84%	96%	3%	1%	99.9%	0.1%
Discharge letters	2830	13%	87%	94%	6%	0%	98%	2%

Table 4 shows the number of English triggers used by the original ConText algorithm and the number of Dutch language triggers used by ContextD. About 60% of the English triggers were translated one-to-one into Dutch. For the remaining English triggers, several possible Dutch translations were added resulting in a much larger number of Dutch triggers. For example, for the English negation trigger ‘never had’, three equivalent Dutch triggers were added: ‘nooit gehad’, ‘had nooit’, and ‘hadden nooit’. All the original triggers from ConText were translated to Dutch without particular issues.

**Table 4:** **Number of English and Dutch trigger terms for each contextual property**

**Contextual property**	**English triggers**	**Dutch triggers**
Negation	160	395
Temporality	42	62
Experiencer	44	52
Total	246	509

Table 5 shows the performance on the evaluation set of the ConText algorithm using only automatically and manually translated Dutch triggers (baseline) and of the ContextD algorithm after all modifications (final). The baseline performance of the algorithm was poor on the historical terms and could not identify a single hypothetical term. The experiencer property was the easiest to assign, which is reflected in the high baseline performance. For the negation property, the precision was high for all report types but the algorithm missed many negated terms, i.e., recall was low. For the final ContextD, the recall was considerably improved for negation and historical values on all report types. Although the performance was improved for hypothetical values on specialist letters and radiology reports, overall it remained poor.

**Table 5:** **Results on the evaluation set using only the translated terms from English to Dutch (baseline) and the final ContextD results with modifications (final)**

		**Precision**		**Recall**		**F-score**	
**Property value**	**Total**	**Baseline**	**Final**	**Baseline**	**Final**	**Baseline**	**Final**
Negated							
GP entries	175	0.96	0.88	0.66	0.90	0.78	0.89
Specialist letters	177	0.93	0.84	0.63	0.90	0.75	0.87
Radiology reports	287	0.96	0.91	0.55	0.97	0.70	0.93
Discharge letters	180	0.98	0.92	0.67	0.93	0.79	0.92
**Recent**							
GP entries	1365	0.97	0.98	0.98	0.94	0.98	0.96
Specialist letters	919	0.91	0.95	0.99	0.92	0.95	0.94
Radiology reports	1341	0.97	0.98	0.98	0.96	0.97	0.97
Discharge letters	1140	0.93	0.97	0.98	0.91	0.95	0.94
**Historical**							
GP entries	28	0.15	0.17	0.17	0.54	0.16	0.26
Specialist letters	66	0.47	0.41	0.10	0.76	0.17	0.54
Radiology reports	52	0.30	0.37	0.30	0.67	0.30	0.48
Discharge letters	90	0.36	0.39	0.13	0.78	0.19	0.52
**Hypothetical**							
GP entries	17	0	0	0	0	0	0
Specialist letters	29	0	0.67	0	0.07	0	0.13
Radiology reports	6	0	0.67	0	0.33	0	0.44
Discharge letters	0	0	0	0	0	0	0
**Patient**							
GP entries	1379	0.98	0.98	1.00	0.99	0.99	0.99
Specialist letters	999	0.99	0.99	1.00	0.99	0.99	0.99
Radiology reports	1398	0.99	1.00	1.00	1.00	1.00	1.00
Discharge letters	1220	0.98	0.99	1.00	1.00	0.99	0.99

The ContextD algorithm utilized 23 unique triggers to identify *negated* terms, 5 unique triggers to identify *historical* terms, 3 unique triggers to identify *hypothetical* terms, and 10 unique triggers to identify *other* terms across all report types. Among the 23 unique triggers for the negation property, the trigger term ‘geen’ (no) was used most frequently. The most used triggers for the temporality property and the experiencer property were ‘status na’ (status after) and ‘moeder’ (mother), respectively. A list of all unique triggers and their frequency of occurrence in the evaluation set is given in the Additional file 1.

Table 6 shows an analysis of 25 randomly selected false negatives for different contextual property values in the evaluation set. In 40% of the errors, the evidence trigger was missing from our trigger list. For instance, in the entry ‘Fam.anamn blanco voor trombose…’ (No family history for thrombosis…) the trigger *blanco voor* was missing, resulting in misclassifying the negated concept *trombose* as Not Negated. These errors can be prevented by adding triggers to the ContextD trigger lists. It is important to note here that some of the trigger terms causing these errors (e.g., *is weg [is gone]*) were intentionally not added in the triggers list to avoid too many false positives. In 19% of the errors, a pre- or post-trigger alone could not correctly identify the property value of a term. These errors may be prevented by rules that combine pre- and post-triggers along with the distance to the actual term (see Combined Triggers) or rules restricting the scope of a particular trigger. For example, in the sentence ‘Ochtendstijfheid: nee Nachtelijk rugpijn: nee, Wel zonne-allergie…’ (Morning stiffness: no nightly back pain: no, sun allergy present…), the concept *Ochtendstijfheid* could have been identified by adding*: nee* as a post-trigger with a maximum scope of 2 words In 17% of the errors, the sentences were too complex to identify and generalize any trigger or pattern. For example, in the sentence ‘flinke ruizen drukpijn colon erge pijn in flank sinds een aantal dagen dacht zelf aan niersteen advies’ (significant wheezing pressure pain colon severe pain in flank since a few days was thinking of kidney stone advise), the concept *niersteen (kidney stone)* is hypothetical. The possible trigger *dacht zelf (thought himself or herself)* could not be used because of its negative impact in terms of false positives. In 8% of the errors, a variation of the trigger (e.g., a different verb form) was used. The remaining 16% of the errors were due to miscellaneous reasons, such as typos (e.g., no space between the trigger and other words), sentence splitting errors, or the trigger being in another sentence than the condition.

**Table 6:** **Error analysis of false negatives in the evaluation set**

**Error**	**Negated**	**Historical**	**Hypothetical**	**Patient**	**Total**
Missing trigger	15	7	7	11	40
Complex trigger	1	8	2	8	19
Complex sentence	1	-	15	1	17
Trigger variation	-	7	-	1	8
Other	9	3	1	4	16
**Total**	25	25	25	25	100

Table 7 shows an analysis of 25 randomly selected false positives for the different property values. The hypothetical and patient values had less than 25 false positives, so all those available were included in the analysis. In 37% of the errors, the scope of the evidence trigger wrongly included the condition. For example, in the sentence ‘Conclusie Geen oogheelkundige verklaring voor de hoofdpijn’ (Conclusion No ophthalmologic explanation for the headache), the pre-trigger *Geen* is wrongly negating the concept *hoofdpijn* although it has a limited scope. Annotation errors caused 14% of the errors. Half these annotation errors were because the annotators failed to pick the historical trigger ‘status na’ (status after) resulting in those terms being labeled as either Recent or Hypothetical*.* Two ambiguous triggers for the experiencer property (‘pa’, which could mean ‘dad’ or ‘pathology’, and ‘oma’, which could mean ‘grandmother’ or ‘acute otitis media’) caused 14% false positives. Some of the regular expressions in our temporality module caused 11% of the errors because they were either not specific enough or were missing some variations in the text. For example, in the sentence ‘… geen dyspnoe wel net influenza gehad ferro en vit c als <3 weken niet beter revisie…’ (…no dyspnea recently had influenza ferro and vit c if <3 weeks not better revision…), the temporality module identified *3 weken (3 weeks)* close to the concept *influenza* and wrongly labeled it as historical. These types of errors could be avoided by looking for extra evidence such as *net (recently)* and relational operators such as < in combination with the time. In 9% of the false positives, the error was due to missing pseudo triggers. For example, in the sentence ‘met requip niet minder krampen en wel zwabberig,…’ (with requip no fewer cramps and also unstable,…), the pseudo-trigger *niet minder* was missing in the trigger list, resulting in wrongly classifying *krampen* as Negated. The remaining 15% errors were due to several other reasons.

**Table 7:** **Error analysis of false positives in the evaluation set**

**Error**	**Negated**	**Historical**	**Hypothetical**	**Patient**	**Total (%)**
Trigger does not apply to condition	9	7	8	8	32 (37)
Annotation error	2	8	2	-	12 (14)
Ambiguous trigger	-	-	-	12	12 (14)
Trigger problem	-	10	-	-	10 (11)
Missing pseudo trigger	8	-	-	-	8 (9)
Other	6	-	3	4	13 (15)
**Total**	25	25	13	24	87 (100)

Table 8 shows a comparison of the performance of the final ContextD algorithm and the original ConText algorithm. The original ConText algorithm was evaluated on six different English clinical document types [13]. For the comparison, we have selected two document types which appear similar in both studies. An absent precision or recall means that the results could not be calculated because the sum of true positives and false positives or the sum of true positives and false negatives was zero [13]. For the negation property, both algorithms have the same F-score for the radiology reports, but ContextD appears to perform somewhat better on the discharge letters. For the historical property, no comparison could be made for the radiology reports since no F-score was provided for the ConText algorithm. For discharge letters, the ConText algorithm performs better. The low performance of ContextD is due to the high number of false positives (low precision) of which many are annotation errors. For the hypothetical property, no comparison on the same document type could be made since for the radiology reports no results were provided for the ConText algorithm, and for the discharge letters no hypothetical terms were present in the Dutch corpus. For the experiencer property, both algorithms performed equally well.

**Table 8:** **Comparison of the original ConText algorithm for English with the adapted ContextD algorithm for Dutch**

		**ConText (English)**			**ContextD (Dutch)**		
**Category**	**Document type**	**Precision**	**Recall**	**F-score**	**Precision**	**Recall**	**F-score**
Negation	Radiology reports	1.00	0.86	0.93	0.91	0.97	0.93
	Discharge letters	0.84	0.89	0.86	0.92	0.93	0.92
Historical	Radiology reports	-	-	-	0.37	0.67	0.48
	Discharge letters	0.68	0.77	0.73	0.39	0.78	0.52
Hypothetical	Radiology reports	-	-	-	0.67	0.33	0.44
	Discharge letters	1.00	0.92	0.96	-	-	-
Experiencer	Radiology reports	-	-	-	1.00	1.00	1.00
	Discharge letters	1.00	1.00	1.00	0.99	1.00	0.99

For ConText, the results are taken from [13]. Only the similar document types in both studies are selected for comparison.

## Discussion

In this paper we describe and evaluate ContextD, an algorithm to identify contextual properties of medical terms in Dutch clinical text. To develop and test ContextD, we have also created the EMC Dutch clinical corpus, with annotations for the three contextual properties negation, temporality, and experiencer.

The EMC Dutch clinical corpus covers four different types of electronically stored clinical text: entries from the general practitioner, radiology reports, and two sets of medical letters after outpatient treatment (i.e. specialists’ letters) or hospital admission (i.e. discharge letters). The combination of these texts can be considered a representative selection of the documented medical process in the broadest sense, including the patient’s first interactions with the general practitioner, referrals and advanced (imaging) diagnostics in the hospital, and ultimately reporting back to the general practitioner after polyclinic consult or discharge after hospital admission.

Although the GP entries have the smallest size among the four document types in our corpus, they contain more UMLS terms than the discharge letters, which are the largest in size. This can be explained by the fact that our Dutch term list was small, containing mainly common clinical terms, which are more likely to be mentioned in GP records. The statistics shown in Table 1, therefore, do not give a realistic view on the occurrence and coverage of clinically relevant terms in different Dutch clinical texts. A more complete Dutch term list would have identified many more terms in the clinical text.

The corpus was annotated by two independent annotators. Looking at the differences between the annotators a few observations can be made. Medically-schooled annotators are prone to using information outside the context and also make considerations based on prior knowledge concerning the natural course of a condition. On various occasions, one annotator labeled a term as historical based on the assumed chronicity of the disease. At times, annotators had different opinions about keywords such as ‘status na’ (status after), which suggests a longer existing condition. One annotator considered such cases as a part of medical history and often labeled the terms as historical whereas the other annotator sometimes labeled the terms as recent and sometimes as historical because of the uncertain time frame. The annotators often differed on the assignment of hypothetical values to terms, e.g., for terms that were part of a differential diagnosis. In the sentence ‘differentiaal diagnostisch werd gedacht aan appendicitis of diverticulitis’ (for the differential diagnosis appendicitis and diverticulitis were considered), one annotator labeled appendicitis and diverticulitis as recent, reasoning that if they exist they exist now, whereas the second annotator labeled both terms as hypothetical. The inter-annotator agreement for ‘Patient’ is low for the radiology reports (cf. Table 2), which can be explained by the very low number of non-patients (class ‘Other’, see Table 3). With such highly imbalanced class distributions, even a small number of annotation disagreements can result in a low kappa value.

ContextD baseline results showed poor performance for ‘Historical’ and ‘Hypothetical’ values (cf. Table 5). The *recent* and *patient* values, which were the default values for the temporality and experiencer properties, showed good results. The final ContextD results (cf. Table 5) show the improvements especially for the negation and historical values. The most difficult category turns out to be the hypothetical value for the GP entries where the algorithm failed to correctly identify a single hypothetical value. Only few hypothetical terms were contained in the corpus, even less in the training set that we used to expand our trigger lists. We did not find many consistent patterns in the training set to identify hypothetical terms effectively. About a third of the errors in the evaluation set were due to the missing trigger ‘bij’ (upon), which did not occur in the training set. The rest of the errors were due to the sentences being too complex to identify and generalize a trigger or a pattern.

Although we had a much larger list of Dutch triggers compared to the English triggers, only a small number of trigger phrases accounted for the majority of the detected terms (see Additional file 1). This finding is consistent with findings in other languages [9,26,37]. Out of 395 possible Dutch triggers for the negation property, only 23 negation triggers were actually found in the evaluation set. The error analysis on the evaluation set suggested a number of new triggers to identify negations, historical, hypothetical, and experiencer property across all report types. Some of these triggers were intentionally not included in the trigger lists because they decreased rather than improved performance on the development set. A similar problem of some triggers negatively affecting the result was also found in the Swedish study [26].

Although some automatic and linguistically motivated approaches exist to detect the scope [17,18,38], the default scopes used in ContextD are approximate due to lack of full grammaticality in the clinical text. Apart from the standard termination triggers, some additional constraints such as punctuations were added to limit the scope of triggers in GP entries and in specialist letters. The scope for negation was varied in length but never extended past the sentence boundary. Thus, negations that stretched over sentence boundaries were missed. The value of contextual properties may depend on the section of the clinical text, e.g., a symptom described in the previous history section will become historical regardless of how it is phrased. This information was not provided to the ContextD algorithm and as a consequence terms may have been wrongly classified. As mentioned above, annotators sometimes used prior medical knowledge concerning the natural course of a condition to label a value of the contextual property, e.g., assigning historical value to a term for which the chronicity is assumed. Finding the right value for such terms is difficult for algorithms like ContextD, which rely solely on the information present in the direct neighborhood of the term. No effort was made in ContextD to separate patient-reported symptoms (complaints) and suspected diagnoses from the actual diagnoses made by the physician. The suspected diagnoses are usually hypothetical whereas symptoms and actual diagnoses are not, a distinction which requires understanding of the text and therefore is difficult to make for ContextD-like approaches. It is also important to note that the ConText algorithm is a simple algorithm meant to identify simple expressions using trigger lists, and was never expected to capture all attributes. We used case-insensitive exact string matching to find the UMLS terms in the documents. Any variation of a term such as a spelling mistake is likely to be missed by this approach. The same can also be true for the trigger terms. It is also to note that the terms with linguistic variability may occur in variable contexts, which may require some adjustments in the trigger scope or in the regular expressions.

The ContextD algorithm showed good performance in identifying negation and experiencer contextual properties. The performance for the historical and hypothetical (and even for negation and experiencer) properties can be further improved by adding new triggers found in the evaluation set. We observed some errors due to sentence splitting with Apache OpenNLP [34], which is trained on regular natural language text. Retraining the sentence splitter to work better with the Dutch clinical text, especially for the GP entries and specialist letters, would resolve some of the issues related to the missing context. The radiology reports and discharge letters are grammatically well structured; therefore, deep sentence parsing and using rule-based or machine-learning techniques to estimate the trigger scopes for these reports can be employed. To determine historical and hypothetical concepts better, it is important to incorporate information about the specific parts of clinical text (e.g., pre-history and diagnosis) in the algorithm. An extended assertion model that supports multiple values of negation is required to deal with speculation, e.g., the disagreements on diseases in the differential diagnosis.

## Conclusions

The ContextD algorithm showed good performance in identifying terms with negations and identifying who has experienced a particular medical condition across all four report types. The temporality property appears to be the most difficult one and methods to identify this property need to be further developed. The anonymized EMC Dutch clinical corpus, which was annotated for the three contextual properties negation, temporality, and experiencer, is the first publically available Dutch clinical corpus and can serve as a useful resource for further algorithm development.

## Additional file

Additional file 1:
**Regular expressions and the rules used in the Temporality module.**
