# Supplementary material for: ContextD: an algorithm to identify contextual properties of medical terms in a Dutch clinical corpus
Source: BMC Bioinformatics. 2014 Nov 29;15(1):373. doi: 10.1186/s12859-014-0373-3 (PMC4264258; doi:10.1186/s12859-014-0373-3)
Supplement: Additional file 1: — Regular expressions and the rules used in the Temporality module. [file 12859_2014_373_MOESM1_ESM.docx]

**Regular expressions and the rules used in the Temporality module**

| Pattern 1 | |
| --- | --- |
| Example | ...(term)...4 weken geleden .... (term) |
| Regular expression | "\\b(\\d+)\\s*(weken\|wk\|maand\|maanden\|jr\|jaar\|jaren)\\s+(geleden\|gel\|gld){0,1}\\b" |
| Rules | - If pattern matches   - If time mentioned is a week (weken or wk)     - check the digits before       - if week > 2         - Count number of words from the start of the regular expression and the marked term in the sentence (on both sides)   If distance < 30 words  Assign ‘Historical’ |

| Pattern 2 |  |
| --- | --- |
| Example | … (term) ..... nu X-X weken.... (term) |
| Regular expression | "nu\\s+(\\d+)[\\s-](\\d+)\\s*(wk\|weken\|maand\|maanden){1}" |
| Rules | - If pattern matches   - If first digit is >= 2     - Count number of words from the start of the regular expression and the marked term in the sentence (on both sides)       - If distance < 30 words         - Assign ‘Historical’ |

| Pattern 3 |  |
| --- | --- |
| Example | ... <overleden aan> ..... (term). .. <time> <leeftijd> .... |
| Regular expression | "(overleden aan)\\s*(.*)\\s*(\\d+)\\s*(wk\|weken\|maand\|maanden\|jr\|jaar\|jaren\|jarige)\\s*([Ll]eeftijd){0,1}" |
| Rules | - If pattern matches   - If number of words between ‘overleden aan’ and ‘leeftijd’ < 30 words     - If marked term present in between these terms       - Assign ‘Historical’ |

| Pattern 4 |  |
| --- | --- |
| Example | … <time> ... (term) ... <gehad> … |
| Regular expression | "(\\d+)\\s*(wk\|weken\|maand\|maanden\|jr\|jaar\|jaren)\\s*(.*)(gehad){1}" |
| Rules | - If pattern matches   - If time mentioned is a week (weken or wk)     - check the digits before       - if week > 2         - if marked term present between ‘time’ and ‘gehad’   Assign ‘Historical’ |

| Pattern 5 |  |
| --- | --- |
| Example | ... <time> ... (term) ... |
| Regular expression | “(vorig[e]{0,1}\|voorgaand[e]{0,1}\|afgelopen\|laatst[e]{0,1})[\\s-_]+(mnd\|maand\|maanden\|jr\|jaar\|jaren)(.*)” |
| Rules | - If pattern matches   - Count number of words between end of time pattern and the marked term in the sentence     - If distance < 30 words       - Assign ‘Historical’ |

| Pattern 6 |  |
| --- | --- |
| Example | … (term) … in <time> … (term) … |
| Regular expression | "[I\|i]n[\\s'-]*(jan\|januari\|feb\|februari\|maart\|mrt\|april\|apr\|mei\|jun\|juni\|juli\|jul\|augustus\|aug\|september\|sept\|sep\|okt\|oktober\|nov\|november\|dec\|december){0,1}[\\s\\.'-]*(19\|20){0,1}(\\d{2})\\b" |
| Rules | - If pattern matches   - Count number of words between the pattern string and the marked term in the sentence, term could be pre or post pattern     - If distance < 30 words       - Assign ‘Historical’ |

| Pattern 7 |  |
| --- | --- |
| Example | … (term) … <time>… (term) … |
| Regular expression | "(jan\|januari\|feb\|februari\|maart\|mrt\|april\|apr\|mei\|jun\|juni\|juli\|jul\|augustus\|aug\|september\|sept\|sep\|okt\|oktober\|nov\|november\|dec\|december){1}[\\s\\.'-]*(19\|20){0,1}(\\d{2})\\b" |
| Rules | - If pattern matches   - Count number of words between the pattern string and the marked term in the sentence, term could be pre or post pattern     - If distance < 30 words       - Assign ‘Historical’ |

| Pattern 8 |  |
| --- | --- |
| Example | … <had> ... (term) ... |
| Regular expression | "\\b(had)\\s+(.*)" |
| Rules | - If pattern matches   - Count number of words between ‘had’ and the marked term in the sentence     - If distance <= 2       - Assign ‘Historical’ |

| Pattern 9 |  |
| --- | --- |
| Example | ... <since XX time> … (term) … |
| Regular expression | “\\b(S\|s)in(ce\|ds)\\s+(\\d+)\\s*(wk\|weken\|maand\|maanden\|jr\|jaar\|jaren)\\s*(.*)” |
| Rules | - If pattern matches   - If time is in weeks     - If week > 2       - Count the number of words between the time and the marked term in the sentence, term could be pre or post pattern         - If distance < 30 words   Assign ‘Historical’ |

| Pattern 10 |  |
| --- | --- |
| Example | … (term) ... <op Date> ... (term) … |
| Regular expression | "(?<preSen>.*)\\b(op)\\s+(\\d{1,2})[\\.-]{1}(\\d{1,2})[.-]{1}(19\|20){0,1}(\\d{2})\\b(?<postSen>.*)" |
| Rules | - If pattern matches   - Count the number of words between date and the marked term in the sentence, term could be pre or post pattern     - If distance < 30       - Assign ‘Historical’ |

| Pattern 11 |  |
| --- | --- |
| Example | ... (term) ... <in het [verre] verleden> ... (term) ... |
| Regular expression | "(?<preSen>.*)\\b(in het){1}\\s+(verre ){0,1}(verleden){1}\\b(?<postSen>.*)" |
| Rules | - If pattern matches   - Count the number of words between ‘in het [verre] verleden’ and the marked term in the sentence, term could be pre or post pattern     - If distance < 30       - Assign ‘Historical’ |

| Pattern 12 |  |
| --- | --- |
| Example | ... (term) ... <op dd-jarige leeftijd> ... (term) ... |
| Regular expression | “(?<preSen>.*)op\\s+(?<Year>[\\d](file:///\\d){1,2})[\\.-]{0,1}\\s*([J\|j]arige leeftijd)[\\b](file:///\\\\b)(?<postSen>.*)” |
| Rules | - If pattern matches   - Count the number of words between the pattern and the marked term in the sentence, term could be pre or post pattern     - If distance < 30       - Assign ‘Historical’ |

| Pattern 13 |  |
| --- | --- |
| Example | ... (term) … <jaar geleden> ... (term) ... |
| Regular expression | "(?<preSen>.*)\\b(jr\|jaar\|jaren)\\s+(geleden\|gel\|gld)\\b(?<postSen>.*)" |
| Rules | - If pattern matches   - Count the number of words between the pattern and the marked term in the sentence, term could be pre or post pattern     - If distance < 30       - Assign ‘Historical’ |

| Pattern 14 |  |
| --- | --- |
| Example | ... <oud> ... (term) ... |
| Regular expression | "(?<preSen>.*)\\b(oud\|oude)\\b\\s+(?<postSen>.*)" |
| Rules | - If pattern matches   - Count the number of words between the pattern and the marked term in the sentence     - If distance <= 3       - Assign ‘Historical’ |

| Pattern 15 |  |
| --- | --- |
| Example | ... (term) ... <in haar/zijn jeugd> ... (term) ... |
| Regular expression | "(?<preSen>.*)\\b([i\|I]n)\\s+(haar\|zijn)\\s+(jeugd)\\b(?<postSen>.*)" |
| Rules | - If pattern matches   - Count the number of words between the pattern and the marked term in the sentence     - If distance < 30       - Assign ‘Historical’ |

| Pattern 16 |  |
| --- | --- |
| Example | .... (term) … <since YEAR> .... (term) … |
| Regular expression | "(?<preSen>.*)\\bsin(ce\|ds)[\\s'-]*(19\|20){0,1}(\\d{2})\\b(?<postSen>.*)" |
| Rules | - If pattern matches   - Count the number of words between the pattern and the marked terms in the sentence     - If distance < 30       - Assign ‘Historical’ |

List of UMLS semantic types used to select Dutch medical terms from the UMLS

- Acquired Abnormality
- Anatomical Abnormality
- Antibiotic
- Biologically Active Substance
- Biomedical or Dental Material
- Carbohydrate
- Cell or Molecular Dysfunction
- Chemical
- Chemical Viewed Functionally
- Chemical Viewed Structurally
- Clinical Drug
- Congenital Abnormality
- Disease or Syndrome
- Eicosanoid
- Element Ion or Isotope
- Experimental Model of Disease
- Finding
- Hazardous or Poisonous Substance
- Hormone
- Immunologic Factor
- Indicator Reagent or Diagnostic Aid
- Injury or Poisoning
- Inorganic Chemical
- Lipid
- Mental or Behavioral Dysfunction
- Neoplastic Process
- Neuroreactive Substance or Biogenic Amine
- Nucleic Acid Nucleoside or Nucleotide
- Organic Chemical
- Organophosphorus Compound
- Pathologic Function
- Pharmacologic Substance
- Sign or Symptom
- Steroid
- Vitamin

Unique triggers used by the ContextD algorithm to identify the values of three contextual properties in the evaluation set

| Negation |  | Historical |  | Hypothetical |  | Experiencer |  |
| --- | --- | --- | --- | --- | --- | --- | --- |
| afwezigheid van (absence of) | 1 | destijds (at the time) | 1 | als er (if there) | 3 | broer (brother) | 3 |
| evenmin (nor) | 4 | medische voorgeschiedenis (medical history) | 1 | als ze (if they) | 1 | grootvader (grandfather) | 1 |
| gedaald (decreased) | 1 | status na (status after) | 79 | indien (if) | 13 | ma (mom) | 1 |
| geen (none) | 575 | VG (history) | 11 |  |  | moeder (mother) | 17 |
| geen aanwijzingen voor (no evidence for) | 98 | voorgeschiedenis (history) | 39 |  |  | oma (grandma) | 2 |
| geen klachten van (no complaints) | 2 |  |  |  |  | opa (grandpa) | 1 |
| geen oorzaak van (no cause for) | 1 |  |  |  |  | pa (dad) | 8 |
| geen teken van (no sign of) | 8 |  |  |  |  | vader (father) | 3 |
| geen tekenen van (no signs of) | 21 |  |  |  |  | zus (sister) | 5 |
| heeft geen (have not) | 5 |  |  |  |  | zuster (sister) | 1 |
| kan niet (can not) | 1 |  |  |  |  |  |  |
| leek niet (seemed not) | 1 |  |  |  |  |  |  |
| niet (not) | 71 |  |  |  |  |  |  |
| niet als (not as) | 1 |  |  |  |  |  |  |
| onbekend (unknown) | 2 |  |  |  |  |  |  |
| uitsluiten (exclude) | 1 |  |  |  |  |  |  |
| verdwenen (disappeared) | 4 |  |  |  |  |  |  |
| vertonen geen (do not exhibit) | 3 |  |  |  |  |  |  |
| vertoonde geen (showed no) | 1 |  |  |  |  |  |  |
| vrij van (free from) | 1 |  |  |  |  |  |  |
| weg (gone) | 3 |  |  |  |  |  |  |
| zonder (without) | 44 |  |  |  |  |  |  |
| zonder tekenen van (without signs of) | 4 |  |  |  |  |  |  |

**Annotation guidelines for EMC ContextD corpus**

The EMC Dutch Clinical Corpus is marked with Dutch clinical terms (conditions or symptoms) that are contained in the UMLS. The job of the annotators is to find the value of three contextual properties for each of these *marked* terms. They are not to label unmarked text. The three contextual properties are: negation, temporality, and experiencer. Following is the description of each of these contextual properties with instructions on how to label them:

1. Negation

This property indicates whether the condition or symptom to which the term refers, is present. It can have two possible values: “Negated” and “Not Negated”. Label the term as ‘Negated’ if there is evidence in the text suggesting that the condition or symptom does not exist or occur, otherwise label the term as ‘Not Negated’. For example:

- ‘The patient denies any *nausea’*

The verb ‘denies’ suggests that the condition ‘nausea’ does not exist. The term will therefore be labeled as ‘Negated’.

- ‘He has also never used medications that affect *tinnitus’*

The negation (never) in this sentence referred to the use of medication, not to the term *tinnitus*. Therefore, the term will be labeled as ‘Not Negated’.

- ‘A knee exam did not show signs of *hydrops* or *atrophy’*

In this sentence both the terms *hydrops* and *atrophy* will be labeled as ‘Negated’.

1. Temporality

This property places a condition along a simple time line. There are three possible values for this property: ‘Recent’, ‘Historical’, and ‘Hypothetical’. Terms are labeled ‘Historical’ if the condition or symptom started more than two weeks before the clinical note was made. If the condition or symptom started less than two weeks ago, the term is labeled ‘Recent’. The value 'Hypothetical' is assigned to terms that are neither ‘Recent’ nor 'Historical', e.g., mentioning of a scenario that might happen in future.

- ‘She is having *cough* and *fever* due to cold’

The terms *cough* and *fever* are labeled as ‘Recent’ since they are currently present.

- ‘Patient has a history of *chest pain’*

The text indicates that the patient has previously suffered from chest pain. He may still have it but it seems likely it started more than two weeks ago. The term will be labeled as ‘Historical’.

- ‘Patient should return if she develops *fever’*

The text suggests that the patient does not have fever at this time, but may have in the future. The term *fever* is labeled as ‘Hypothetical’.

- ‘The patient is referred for a CT scan to screen for *lung cancer*’

The text suggests that the physician is suspecting that the patient may have lung cancer. The term *lung cancer* is labeled as ‘Hypothetical’ since lung cancer is not yet confirmed.

Note: For a ‘Hypothetical’ term the Negation should be set to ‘Not Negated’.

1. Experiencer

This property describes whether the patient or someone else experiences the condition.

It can have two values: ‘Patient’ or ‘Other’. It is assumed that a clinical term refers to the patient, unless explicitly specified otherwise. For example:

- ‘The patient’s father has a history of *renal disease*’

The text clearly suggests that the term *renal disease* is referring to the father of the patient, and not to the patient. Therefore, the experiencer property will be labeled as ‘Other’.

- ‘Examination of the right foot shows that there is no *hallux valgus*’

The text does not explicitly suggest that someone other than the patient was examined. Therefore, the experiencer property will be labeled as ‘Patient’.
